# Determining the degree of adherence to treatment in inflammatory bowel disease patients

**Published:** 2018

**Authors:** Hedieh Balaii, Sepideh Olyanasab Narab, Binazir Khanabadi, Fakhri Alsadat Anaraki, Shabnam Shahrokh

**Affiliations:** 1 *Basic and Molecular Epidemiology of Gastrointestinal Disorders Research Center, Research Institute for Gastroenterology and Liver Diseases, Shahid Beheshti University of Medical Sciences, Tehran, Iran*; 2 *Gastroenterology and Liver Diseases Research Center, Research Institute for Gastroenterology and Liver Diseases, Shahid Beheshti University of Medical Sciences, Tehran, Iran*; 3 *Foodborne and Waterborne Diseases Research Center, Research Institute for Gastroenterology and Liver Diseases, Shahid Beheshti University of Medical Sciences, Tehran, Iran.*; 4 *Colorectal Division of Surgical Ward, Taleghani Hospital, Shahid Beheshti University of Medical Science, Tehran, Iran*

**Keywords:** Inflammatory bowel disease, Ulcerative colitis, Crohn’s disease, Adherence, Quality of life

## Abstract

**Aim:**

Since the inflammatory bowel disease (IBD) is a disorder which requires continuous drug intake to induce and maintain the remission phase, finding the barriers for low adherent group, may improve the disease phase and quality of life in those patients.

**Background:**

IBD is defined as a chronic immune disorder with unpredictable flares. The common treatment of these diseases can be effective for inducing and maintaining remission courses. Therefore the use of long-term medication therapy is the crucial key to prevent surgery and complications in patients with IBD.

**Methods:**

Morisky Medication Adherence Scales (MMAS) is used for detecting level of adherence to the medicines for 137 patients with IBD. Demographic and clinical data are recorded for all patients and quality of life has been evaluated by Short-Form 36 questionnaire in 55 patients.

**Results:**

Demographic and clinical features showed no correlation with the degree of adherence. The MMAS-8 score in the low adherent group significantly different than that in the medium and high adherer group. No relation was found statistically between level of adherence and mental or physical sates (*p* value=0.17, 1.2) and total quality of life (*p* value=0.22) in patients with IBD.

**Conclusion:**

Designing smart reminder and the physician’s explain about adverse effects and beneficial of medicines may be effective to confront with forgetfulness and feeling comfort with treatment. Improve a strategy in order to regular measurement of adherence to medication, provides enough information about stop taking medication.

## Introduction

 The inflammatory bowel disease (IBD) encompassing Crohn’s disease (CD) and ulcerative colitis (UC) is defined as chronic immune disorders with unpredictable flare (1, 2). The common treatment of these diseases: mesalamine-based compounds, corticosteroids, thiopurines, and anti-TNF therapies can be effective for inducing and maintaining remission (3, 4). Therefore, the use of long-term medication therapy is the crucial key to prevent surgery and complications in patients with IBD. Poor management of disease may contribute to low adherence associated with increasing the risk of relapses, colorectal cancer and eventually mortality and a decreased quality of life (5-7). Low adherence to medication has been reported in previous studies rating 40-72% in individuals suffering from IBD, resulting exacerbations of the disease (3, 5, 7-10). A high adherence would be achievable by enough information about continuous therapy and follow-ups due to a good cooperation between physician and patient (11, 12). Identifying the barriers leading to non-adherence to the drugs, can address to provide appropriate educational needs for IBD patients (13, 14).

The term “compliance” has been expressed by Sackett and Haynes, which is associated with patient’s obedience to prescribed medicine and improved regimens and quality of life (15-17). On the other hand the exact goal of drug submission has been proposed by the term “adherence”, which is used to explain a deeper meaning of follow-up than compliance (18). The aim of this study was to determine the accurate measurements of adherence in IBD patients in order to seek the factors which accompany the flare-ups episodes. 

## Methods

The study protocol approval has been confirmed by the Ethics Committee of Shahid Beheshti Medical University. The participants were included to this study, if:

i. they had a diagnosis of IBD approved by clinical, endoscopic, radiologic, and histopathologic criteria (19); 

ii. they were receiving medicine for their IBD by a gastroenterologist; 

iii. they were able to use oral drugs for IBD 

iv. They took medicine for IBD up to six months. 

The patients younger than age 15 and new patients are excluded from the study. Morisky Medication Adherence Scales (MMAS), an 8-question questionnaire, has been validated and used for obtaining level of adherence to the medications (20). Each question scored one point if the answer was “no” in questions 1–4 and 6–7, while the answer “yes” in question 5 added-up one point. The question 8 is scored regarding to the four type of answers (rarely/never: 1.00; once in a while: 0.75; sometimes: 0.50; usually: 0.25; always: 0). The MMAS scores were categorized previously into the following three levels of adherence: high adherence (score: 8), medium adherence (score: 6-7), and low adherence (score: fewer than 6) (21).

In addition, another questionnaire has been designed in order to gather demographic and clinical data, comprising age, gender, marital status, educational level, type of IBD, duration of disease, time for therapy, surgery related to IBD, type of medication, disease phase and behavior according to the Harvey-Bradshaw Index (HBI) disease activity scale for all patients. Quality of life has been evaluated by Short-Form 36 questionnaire (SF-36) in fifty-five patients. 

Statistical analysis was calculated using Kruskal Wallis and One-Way ANOVA tests with p≤0.05 considered significant. All calculations were performed using SPSS 21.0 for Windows (SPSS, Inc, Chicago, IL). 

## Results

A total of 137 patients with IBD (35 CD, 102 UC) participated in the study. The characteristics of these patients are described in Table 1. Different factors such as gender, age, education, marital status, medication, disease behavior, time since diagnosis of IBD, or duration of disease and colectomy did not correlate with the degree of adherence. Also, according to our data, having CD tends to increase the therapeutic adherence to drugs, but these results were not statistically significant. There was also no association between non-adherence and disease type (p= 0.69), and clinical activity of disease in UC and CD patients (p= 0.30, 0.21) (Table 2). 

Based on MMAS-8 responses, we identified 63 (46%) patients as low adherers and 57 (41.6%) patients as medium and 17 (12.4%) high adherers. The MMAS-8 score in the low adherer group was significantly different from that of medium and high adherer group (*p* < 0.001). 

Fifty-five SF-36 questionnaires were answered by patients, but no relation was found statistically between level of adherence and mental or physical states (*p* value=0.17, 1.2) and total quality of life (*p* value=0.22) in patients with IBD.

The main reasons for non-adherence included forgetfulness 42 (50.6%) and feeling hassled about treatments 48 (57.8%).

**Table 1:** Characteristics of 137 patients with IBD

**Characteristics **		N = 137 (%)
Diagnosis	Ulcerative Colitis	102 (74.5)
	Crohn’ s Disease	35 (25.5)
Age (years)	Mean ± SD	33.30 ± 11.66
	Minimum, maximum	15 , 74
	≤30	66 (48.2)
	>30	71 (51.8)
Gender (%)	Male	40 (48.2)
	Female	71 (51.8)
Marital status	Single	57 (41.6)
	Married	78 (56.9)
	Divorced	2 (1.5)
Education	Illiterate	1 (0.7)
	Primary School	14 (10.2)
	High School	56 (40.9)
	Bachelor of Sciences	38 (28.7)
	Master of Sciences	11 (8)
	MD., PhD.	4 (2.9)
	Other	12 (8.8)
Disease behavior	Proctitis	13 (9.5)
	Left sided colitis	21 (15.3)
	Pancolitis	36 (26.3)
	UC + PSC	10 (7.3)
	UC Remission	37 (27.2)
	Penetrating CD	12 (8.8)
	Strictural CD	3 (2.2)
	Inflammatory CD	14 (10.2)
	Penetrating+ Strictural CD	3 (2.2)
	CD Remission	11 (8.1)
Medications	5-ASA	39 (28.5)
	Immunosuppressant	2 (1.5)
	Anti-TNF	6 (4.4)
	5-ASA+ Immunosuppressant	38 (27.7)
	5-ASA+ Anti-TNF	16 (11.7)
	Immunosuppressant+ Anti-TNF	4 (2.9)
	5-ASA+ Immunosuppressant+ Anti-TNF	31 (22.6)
Surgery	UC Colectomy	4 (2.9)
	CD Colectomy	1 (0.7)
Time from onset	<1 yeas	18 (13.1)
	1-5 years	43 (31.4)
	>5 years	39 (47)
Time from diagnosis	<1 yeas	26 (19)
	1-5 years	47 (34.3)
	>5 years	70 (51.1)

**Table 2:** Correlations between adherence degrees and participant endorsed barriers to medication adherence

		low-adherer	medium-adherer	high-adherer	p-value
UC CD		46 (45.1) 17 (48.6)	44 (43.1) 13 (37.1)	12 (11.8) 5 (14.3)	0.80
Age ≤30 Age >30		32 (48.5) 31 (43.7)	26 (39.4) 31 (43.7)	8 (12.1) 9 (12.7)	0.84
Male Female		27 (38.0) 36 (54.5)	36 (50.7) 21 (31.8)	8 (11.3) 9 (13.6)	0.07
Single Married Divorced		27 (47.4) 35 (44.9) 1 (50.0)	23 (40.4) 34 (43.6) 0	7 (12.3) 9 (11.5) 1 (50.0)	0.50
Illiterate Primary School High School Bachelor of Sciences Master of Sciences Other MD, PhD		1 (100) 6 (42.9) 29 (51.8) 16 (42.1) 6 (54.5) 4 (33.3) 1 (25)	0 6 (42.9) 19 (33.9) 19 (50) 4 (36.4) 6 (50) 2 (50)	0 2 (14.3) 8 (14.3) 3 (7.9) 1 (9.1) 2 (16.7) 1 (25)	0.91
Proctitis Left sided colitis Pancolitis Penetrating CD Strictural CD Inflammatory CD Penetrating+ Strictural CD		8 (61.5) 6 (28.6) 19 (52.8) 5 (41.7) 1 (33.3) 7 (50) 1 (33.3)	4 (30.8) 12 (57.1) 16 (44.4) 5 (41.7) 1 (33.3) 5 (35.7) 2 (66.7)	1 (7.7) 3 (14.3) 1 (2.8) 2 (16.7) 1 (33.3) 2 (14.3) 0	0.62
UC+PSC		5 (50)	3 (30)	2 (20)	0.42
UC	Flare-up Remission	32 (31.7) 14 (13.9)	27 (26.7) 17 (16.8)	5 (5.0) 6 (5.9)	0.30
CD	Flare-up Remission	10 (28.6) 7 (20)	9 (25.7) 4 (11.4)	5 (14.3) 0	0.21
5-ASA IM Anti-TNF 5-ASA+ IM 5-ASA+ Anti-TNF IM+ Anti-TNF 5-ASA+ IM+Anti-TNF		20 (51.3) 0 2 (33.3) 20 (52.6) 8 (50) 1 (25) 11 (35.5)	14 (35.9) 2 (100) 1 (16.7) 16 (42.1) 7 (43.8) 2 (50) 15 (48.4)	5 (12.8) 0 3 (50) 2 (5.3) 1 (6.3) 1 (25) 5 (16.1)	0.18
UC Colectomy		0	4 (4.0)	0	0.06
CD Colectomy		1 (2.9)	0	0	0.58
Time from Onset	<1 yeas 1-5 years >5 years	6 (33.3) 20 (46.5) 60 (45.8)	10 (55.6) 18 (41.9) 55 (42)	2 (11.1) 5 (11.6) 16 (12.2)	0.77
Time from Diagnosis	<1 yeas 1-5 years >5 years	8 (30.8) 23 (48.9) 29 (50)	14 (53.8) 19 (40.4) 22 (37.9)	4 (15.4) 5 (10.6) 7 (12.1)	0.55

## Discussion

A successful treatment accompanies with a good cooperation between physician and patient, in order to achieve the best way to control the disease. The more training of the patient regarding the correct use of the drugs and treatment follow-ups would be more desirable. Therefore, seeking obstacles associated with non-adherence may present better solutions for physicians and assistants to improve appropriate drug usage.

The rate of adherence is significantly low in our country comparing to other populations. (2, 8, 22-24)

In some studies a significant correlation is found between degree of adherence and age, gender, educational level, stable affective relationship, types of treatment, disease type, extension and severity, duration of disease and onset and history of colectomy.(18, 22, 25, 26) But in our data, no association revealed between adherence level and mentioned features in accordance with other experience. (10, 27, 28)

Moreover, depression which is known as low adherence trigger in some investigations (18, 23), as well as physical quality of life in patients with IBD do not seem to be impressive in our study. 

The standpoint of barriers associated with low-adherents were feeling hassled about treatments and forgetfulness, respectively similar to other authors (29-31). In summary, we resulted a low rate of adherence in our IBD patients associated with forgetfulness and feeling dissatisfied during a long time. Therefore, designing smart reminder on patient’s phone would be effective to confront forgetfulness. In addition, the physician should spend more time on investigation, so that the patient gets more information about adverse effects of drugs and may be convinced that remission phase is dependent on regular treatment. Moreover, regular evaluation of adherence to medication would be beneficial to follow-up. 

Furthermore, assessment of a comprehensive system with more accurate questions to measure the adherence between IBD patients may improve the knowledge of adherence.

## Conflict of interests

The authors declare that they have no conflict of interest.
